# Complete Genome Sequences of *Pseudomonas monteilii* SB3078 and SB3101, Two Benzene-, Toluene-, and Ethylbenzene-Degrading Bacteria Used for Bioaugmentation

**DOI:** 10.1128/genomeA.00524-14

**Published:** 2014-05-29

**Authors:** Morten S. Dueholm, Mads Albertsen, Seth D’Imperio, Vaibhav P. Tale, Derrick Lewis, Per Halkjær Nielsen, Jeppe Lund Nielsen

**Affiliations:** a Center for Microbial Communities, Department of Biotechnology, Chemistry, and Environmental Engineering, Aalborg University, Aalborg, Denmark; b Novozymes Biologicals, Salem, Virginia, USA

## Abstract

*Pseudomonas monteilii* SB3078 and SB3101 are benzene-, toluene-, and ethylbenzene-degrading strains used for bioaugmentation in relation to treatment of wastewater contaminated with petrochemical hydrocarbons. Complete genome sequencing of the bioaugmentation strains confirms that they are very closely related (100.0% average nucleotide identity). Both strains contain extensive integration of phage elements, with the main difference being insertion of additional phage elements in the SB3078 genome.

## GENOME ANNOUNCEMENT

Members of the gammaproteobacterial genus *Pseudomonas* are Gram-negative, rod shaped bacteria renowned for their remarkable metabolic capacity and physiologic versatility, which enables them to colonize a wide range of environmental niches (1). Strains of *Pseudomonas* are commonly used for biodegradation due to their catabolic repertoire and ability to withstand harsh environmental conditions (2, 3).

*Pseudomonas monteilii* SB3078 and SB3101 are components of BioRemove 2300, a bioaugmentation product made by Novozymes Biologicals (Salem, VA, USA) for enhanced hydrocarbon biodegradation in refining and petrochemical wastewater systems. The strains were isolated from motor oil-contaminated soil (Salem, VA, USA, June 1992) based on their ability to grow on toluene as the sole carbon source. Taxonomic assignment to *P. monteilii* was based on 16S rRNA gene nucleotide sequence analysis, as well as physiological and biochemical features (4).

Genomic DNA was isolated using the PowerMicrobial Maxi DNA isolation kit (MoBIO, Carlsbad, CA, USA). Paired-end and mate-pair libraries were prepared with the Nextera DNA and mate-pair sample preparation kits (Illumina, Germany), respectively. Mate-pair libraries were prepared without any size selection. All procedures were carried out as recommended by the manufacturer. Sequencing of the paired-end and mate-pair libraries was performed using HiSeq2000 and MiSeq sequencers (Illumina, Germany), respectively. The reads were assembled *de novo* using the build-in tool of CLC Genomics 6.0. The average coverage of the assemblies was 162× and 295× for SB3078 and SB3101, respectively. Manual scaffolding of contigs were carried out based on paired-end and mate-pair information. Cytoscape v2.8.3 (5) and Circos (6) were used for visualization and manual inspection of the assemblies as described by Albertsen et al. (7). Gaps were closed by manual read mapping in CLC Genomics 6.0. Annotation was done using the RAST auto-annotation server (8) and the NCBI prokaryotic genome automatic annotation pipeline (PGAAP) (9).

The complete genome sequences are 6,000,087 (SB3078) and 5,945,120 bp (SB3101) with G+C contents of 62.5% (SB3078) and 62.5% (SB3101). The bioaugmentation strains have 100.0% average nucleotide identity (ANIb) and they should consequently be considered variants of the same strain (10). The strains share 98.8% ANIb with their closest completely genome sequenced neighbor *P. putida* S16. Annotation by the NCBI PGAAP identified 5,436 (SB3078) and 5,360 (SB3101) coding sequences (CDS).

Subsystem analysis of the genomes by RAST confirmed the absence of the virulence associated type-III protein secretion system and rhamnolipid synthesis operon found in *P. aeruginosa* (1). The ability to degrade benzene, toluene, and ethylbenzene is encoded by a toluene degradation (TOD) operon identical to that found in *P. putida* F1 (11). Both genomes contained curli and Fap amyloid operons as well as operons for alginate synthesis (12–14). These features allow the bacteria to form biofilms, which are important for their survival in hostile environments.

### Nucleotide sequence accession numbers.

These whole-genome sequences of *P. monteilii* SB3078 and SB3101 were deposited at DDBJ/EMBL/GenBank under the accession numbers CP006978 and CP006979, respectively.

## References

[1] Silby MW Winstanley C Godfrey SAC Levy SB Jackson RW 2011 *Pseudomonas* genomes: diverse and adaptable. FEMS Microbiol. Rev. 35:652–680. 10.1111/j.1574-6976.2011.00269.x 21361996

[2] Thompson IP Van Der Gast CJ Ciric L Singer AC 2005 Bioaugmentation for bioremediation: the challenge of strain selection. Environ. Microbiol. 7:909–915. 10.1111/j.1462-2920.2005.00804.x 15946288

[3] Mrozik A Piotrowska-Seget Z 2010 Bioaugmentation as a strategy for cleaning up of soils contaminated with aromatic compounds. Microbiol. Res. 165:363–375. 10.1016/j.micres.2009.08.001 19735995

[4] Elomari M Coroler L Verhille S Izard D Leclerc H 1997 *Pseudomonas monteilii* sp. nov., isolated from clinical specimens. Int. J. Syst. Bacteriol. 47:846–852. 10.1099/00207713-47-3-846 9226917

[5] Smoot ME Ono K Ruscheinski J Wang P-L Ideker T 2011 Cytoscape 2.8: new features for data integration and network visualization. Bioinformatics 27:431–432. 10.1093/bioinformatics/btq675 21149340 PMC3031041

[6] Krzywinski M Schein J Birol I Connors J Gascoyne R Horsman D Jones SJ Marra MA 2009 Circos: an information aesthetic for comparative genomics. Genome Res. 19:1639–1645. 10.1101/gr.092759.109 19541911 PMC2752132

[7] Albertsen M Hugenholtz P Skarshewski A Nielsen KL Tyson GW Nielsen PH 2013 Genome sequences of rare, uncultured bacteria obtained by differential coverage binning of multiple metagenomes. Nat. Biotechnol. 31:533–538. 10.1038/nbt.2579 23707974

[8] Aziz RK Bartels D Best AA DeJongh M Disz T Edwards RA Formsma K Gerdes S Glass EM Kubal M Meyer F Olsen GJ Olson R Osterman AL Overbeek RA McNeil LK Paarmann D Paczian T Parrello B Pusch GD Reich C Stevens R Vassieva O Vonstein V Wilke A Zagnitko O 2008 The RAST server: Rapid Annotations using Subsystems Technology. BMC Genomics 9:75. 10.1186/1471-2164-9-75 18261238 PMC2265698

[9] Angiuoli SV Gussman A Klimke W Cochrane G Field D Garrity GM Kodira CD Kyrpides N Madupu R Markowitz V Tatusova T Thomson N White O 2008 Toward an online repository of standard operating procedures (SOPs) for (meta)genomic annotation. Omics 12:137–141. 10.1089/omi.2008.0017 18416670 PMC3196215

[10] Richter M Rosselló-Móra R 2009 Shifting the genomic gold standard for the prokaryotic species definition. Proc. Natl. Acad. Sci. U. S. A. 10.1073/pnas.0906412106 PMC2776425 19855009

[11] Cho MC Kang D-O Yoon BD Lee K 2000 Toluene degradation pathway from *Pseudomonas putida* F1: substrate specificity and gene induction by 1-substituted benzenes. J. Ind. Microbiol. Biotechnol. 25:163–170. 10.1038/sj.jim.7000048

[12] Dueholm MS Albertsen M Otzen D Nielsen PH 2012 Curli functional amyloid systems are phylogenetically widespread and display large diversity in operon and protein structure. PLOS One 7:e51274. 10.1371/journal.pone.0051274 23251478 PMC3521004

[13] Dueholm MS Otzen D Nielsen PH 2013 Evolutionary insight into the functional amyloids of the pseudomonads. PLOS One 8:e76630. 10.1371/journal.pone.0076630 24116129 PMC3792158

[14] Franklin MJ Nivens DE Weadge JT Howell PL 2011 Biosynthesis of the *Pseudomonas aeruginosa* extracellular polysaccharides, alginate, Pel, and Psl. Front. Microbiol. 2:167. 10.3389/fmicb.2011.00167 21991261 PMC3159412

